# Coping with the COVID‐19 pandemic through institutional trust: Rally effects, compensatory control, and emotions

**DOI:** 10.1111/ssqu.13002

**Published:** 2021-08-19

**Authors:** Michele Roccato, Pasquale Colloca, Nicoletta Cavazza, Silvia Russo

**Affiliations:** ^1^ Department of Psychology University of Torino Torino Italy; ^2^ Department of Education Studies “Giovanni Maria Bertin” University of Bologna Bologna Italy; ^3^ Department of Communication and Economics University of Modena and Reggio Emilia Reggio Emilia Italy

**Keywords:** compensatory control, emotions, COVID‐19, institutional trust, rally effects

## Abstract

**Objective:**

Exogenous shocks trigger rally effects, leading the public opinion toward increased trust in institutions. Rally effects have an important social function because they help society react to shocks rapidly and efficiently as a single unit and cohesively face the threat. However, little is known about the individual functions that these effects fulfil. In this study, we aimed to analyze the individual functions of the rally effect.

**Method:**

In a three‐wave longitudinal study on a quota sample of the Italian adult population (*N* = 1070), we analyzed the individual consequences of the increase in institutional trust triggered by the COVID‐19 pandemic.

**Results:**

A path analysis showed that increased trust in political, *super partes* and international institutions fostered well‐being, reducing anxiety, collective angst, and anger via the mediation of participants’ perceived control over their lives.

**Conclusion:**

Beyond their well‐known social functions, rally effects fulfil the individual function of favoring people's psychological well‐being.

During the first peak of the COVID‐19 pandemic in Italy, when the numbers of ill people and deaths were growing exponentially, the mass media broadcasted shocking pictures of heaps of coffins piled in the Bergamo Cathedral and dozens of military trucks full of coffins queuing in the highways. At that time, it was undoubtedly touching to see hundreds of people under lockdown singing these words together from their windows and balconies. In this study, using data collected before and during the COVID‐19 pandemic in Italy, we study whether rally effects can be considered as effective coping strategies to face the COVID‐19 shock.

It is well‐known that exogenous shocks lead U.S. public opinion toward increased trust in the current president (Mueller, 1970). When facing a severe threat, U.S. citizens react to the crisis by putting aside their dissatisfaction with the incumbent president, their frustrations and grievances toward the government, and their pre‐existing political differences (Parker, 1995). The consequence is “a unity with the ‘central’ values, the political processes, the moral integrity of the political system, a loyalty to and support of the going order” (Lane, 1962: 162). This “rally effect” is typically triggered by crises in which an enemy, easily identified as evil, confronts a nation as a whole as it happens in wars (Aday, 2010) and terrorist attacks (Schubert, Stewart, and Curran, 2002). In such cases, the media (Oneal and Bryan, 1995) and opposition political leaders (Brody and Shapiro, 1989) reduce their criticism of the governing institutions. From a social‐psychological framework, people are pushed to flow toward a source of social identity (e.g., being Americans) superordinate to that they use when thinking about politics (e.g., being Democrats). Thus, citizens’ differences disappear and their patriotism is heightened (Brewer and Brown, 1998).

Three recent developments in this stream of research are particularly relevant for our study. First, some researchers have shown that rally effects can be generalized outside the U.S. context. Rally effects have been observed in Spain (Dinesen and Jaeger, 2013), Belgium (Kuehnhanss, Holm, and Mahieu, 2021), Japan (Kobayashi and Katagiri, 2018), and South Korea (Hwang, Cho, and Wiegand, 2018). Second, it has been shown that even exogenous crises not triggered by visible enemies who can easily be categorized as evil can generate rally effects. Emblematically, Bol et al. (2020) showed that the COVID‐19 emergency, triggered by an invisible and morally neutral virus, led to a widespread rise in citizens’ trust in national governments across 15 European countries. Esaiasson et al. (2021) found the same result in Sweden, a country characterized by uncommonly high levels of pre‐pandemic institutional trust. Using a sophisticated longitudinal model, Schraff (2020) showed that the increased trust in government exhibited during the COVID‐19 pandemic was a genuine rally effect and not a mere side effect of the lockdown measures implemented by the governments. Finally, exogenous shocks can foster people's institutional trust even beyond their specific government or president. For instance, using panel data, Cavazza et al. (submitted) showed that Italians’ trust in political and *super partes* institutions rose between May and June 2019 (before the COVID‐19 pandemic) and April 2020 (when the pandemic was at its first peak), while trust in international institutions did not. Building on Gorman and Seguin's (2018) idea that exogenous shocks tend to activate the salience of the most relevant portion of people's social identities, these authors suggested that Italians heightened their trust in national–but not international–institutions because the COVID‐19 crisis led to increased salience of national identification and decreased salience of supranational identification.

The literature converges on the idea that “rally around the flag” effects, marked by an increased support for institutions or political leaders in times of crisis, have relevant social functions because they help society react to exogenous shocks rapidly and efficiently as a single unit and cohesively face the threat (Chatagnier, 2012). A verse of the Italian anthem reads “*Stringiamci a coorte, Italia chiamò*” (Let us join in a cohort, Italy has called). However, less is known about the individual functions of rally effects. Does the strengthening of trust in institutions also serve a psychological function in successfully coping with a dramatic event? In this study, building on the literature on compensatory control and coping strategies, we theorized and tested a three‐step longitudinal model of the individual consequences of the rise in institutional trust during the COVID‐19 pandemic.

## Exogenous shocks, compensatory control, and coping

During dramatic exogenous shocks, such as the COVID‐19 pandemic, people tend to experience a perceived loss of control over their lives (Landau et al., 2015). This is problematic because such a perception powerfully influences psychological well‐being (Janoff‐Bulman, 1989). Kay et al. (2011) showed that people may cope with the loss of perceived control by relying on secondary control sources. Overall, religion (Sibley and Bulbulia, 2012), the government (Oneal, Lian, and Joyner, 1996), and anti‐democratic authorities (Mirisola et al., 2014) are powerful sources of secondary control, which can be activated to cope with various threats, ranging from natural (Russo et al., 2020) and climate (Zapata, 2018) disasters to economic crises (Chen, 2010). The same holds true for the threat of the COVID‐19 pandemic (Roccato et al., 2020).

In this study, we reasoned that rally effects can be considered strategies for coping with the weakening of perceived control by resorting to sources of compensatory control. Coping strategies can be functional or not because the actions taken by individuals to face their discomfort are not always actually helpful in achieving that goal (Stoeber and Janssen, 2011). Consistent with the literature above, we expected rally effects to be functional coping strategies to support individuals’ perceived control over their lives and temper their negative emotions. We tested these ideas using a longitudinal design that examined mediation effects in three waves of panel data collected from a quota sample of the Italian population in May–June 2019 (T_1_), April 2020 (T_2_), and October 2020 (T_3_).

## Hypotheses

We expected that increased trust in political, *super partes* and international institutions following the COVID‐19 pandemic would be associated with higher perceived control over their lives (H1). Although trust in international institutions did not increase between T_1_ and T_2_ on a sample level (cf. Cavazza et al., submitted), it is plausible that at the individual level, it may act as a functional coping strategy. We also expected participants’ perceptions of control to be reflected in diminished anxiety (H2), collective angst (i.e., the concern for the ingroup's future vitality experienced when people perceive that a threat is likely to severely harm the ingroup and have difficulty imagining effective ways to protect it: see Wohl and Branscombe, 2009) (H3), and anger (H4). Previous studies have shown that these emotions are typical consequences of exogenous shocks (Lambert et al., 2010).

## METHOD

### Participants

We pursued our research goals by analyzing the longitudinal data set developed within the consequences of COVID‐19 project. The data collection was carried out in accordance with the ethical standards of the Italian Association of Psychology. A wide quota panel of the Italian adult population–stratified by gender, age, geographical area of residence, and size of the area of residence–was surveyed online three times: (a) May 26–June 1, 2019 (T_1_; *N* = 1504); (b) April 17–26, 2020, at the peak of the first wave of the COVID‐19 pandemic (T_2_; *N* = 1195), when Italians were under strict lockdown and were not allowed to leave their homes except to shop for food and medical supplies nearby unless they were essential workers; and (c) October 9–28, 2020 (T_3_; *N* = 1151), when the second wave of the COVID‐19 pandemic was at its onset, managed by the government via lockdown strategies milder than those adopted to tackle the first wave. We had valid data on all of the variables we used for 1070 participants (women = 51.2 percent; *M*_age_ = 50.39, *SD* = 14.31). A logistic regression aimed to predict having ( = 1) versus not having ( = 0) participated in all of the three waves showed no effects of participants’ gender, education, or institutional trust at T_1_ and T_2_. Only age was positively related to survey participation (*B* = 0.02, *SE* = 0.01, *p* < 0.001). However, a low Cox and Snell pseudo‐*R* ^2^ value (0.03) indicated that our panel showed non‐substantial attrition.

### Measures

#### Trust in institutions

In both the first and second waves, we assessed participants’ trust in three types of institutions using nine items borrowed from the European Social Survey (see www.europeansocialsurvey.org), which, respectively, assessed trust in political institutions (political parties, trade unions, parliament, and local administrations; *α*_T1_ = 0.84, *α*_T2_ = 0.85); *super partes* national institutions (the president of the Republic, the judiciary, and the police; *α*_T1_ = 0.73, *α*_T2_ = 0.75); and international institutions (the European Union and the United Nations; *α*_T1_ = 0.82, *α*_T2_ = 0.79). Since we used an 11‐category format in the first wave and a 10‐category format in the second, we rescaled all items to a 0–1 range before averaging them.

#### Perceived control

In the second wave, we measured participants’ perceived control over their lives via the following European Values Survey 10‐category item: “Some people feel they have completely free choice and control over their lives, and other people feel that what they do has no real effect on what happens to them. Please indicate how much freedom of choice and control you feel you have over the way your life turns out.” The 1 and 10 categories were labeled “Not at all” and “A great deal,” respectively.

#### Dependent variables

Following Marcus et al. (2000), we measured participants’ anxiety and anger by asking them to report how often in the days preceding the survey they had felt, respectively, (a) anxiety, fear, and worry (*α* = 0.83), and (b) anger, bitterness, and resentment (*α *= 0.80). Response options were labeled “Never,” “Seldom,” “Often,” and “Always or nearly always” (*α* = 0.83). Based on Wohl and Branscombe (2009), we assessed collective angst using the following four‐category items: “I am worried that the Italian way of life is in jeopardy due to COVID‐19” and “I think the future of the Italian way of life is under threat from COVID‐19” (*α *= 0.84). Response options were labeled “Strongly disagree,” “Disagree,” “Agree,” and “Strongly agree.” We measured all the dependent variables at T_3_ and computed participants’ scores as the mean of these groups of items.

### Data analyses

**FIGURE 1:**
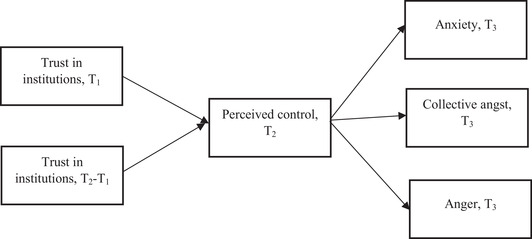
The models tested

Using Mplus 8, we performed three path analyses, one for each group of institutions as summarized in Figure 1. The core variable in the analyses was the difference in trust in institutions between T_1_ and T_2_, which operationalized the rally effect net of the baseline, that is, of trust in institutions at T_1_. We evaluated the fit of our models using the comparative fit index (CFI) and the standardized root mean square residual (SRMR). We considered the models to be satisfactory with a CFI value > 0.90 and an SRMR value < 0.08 (Xia and Yang, 2019).

## RESULTS

The descriptive statistics for the variables we used and their bivariate correlations can be found in the Supplementary Appendix. Table 1 reports the results of our three path analyses, which analyze the associations between increased trust in political institutions, *super partes* institutions, and international institutions and anxiety, collective angst, and anger via the mediation of perceived control. All models had good fit indices. In all models, we found that both institutional trust at T_1_ and the increase in institutional trust between T_1_ and T_2_ were positively associated with perceived control. As expected, we also found that perceived control was linked to low anxiety, collective angst, and anger. The indirect associations between changes in institutional trust and emotions via the mediation of perceived control were negative and significant, with the exception of the indirect association between the change in *super partes* institutions and collective angst, which just approached statistical significance (see Table 2).

**TABLE 1:** Influence of the increase in trust in political institutions on emotions, via the mediation of perceived control (standardized coefficients)

	Trust in political institutions		Trust in *Super Partes* institutions		Trust in international institutions	
**Mediator**:	Perceived control		Perceived control		Perceived control	
	β	*p*	β	*p*	β	*p*
Trust T_1_	0.26	< 0.001	0.17	< 0.001	0.18	< 0.001
Trust change	0.14	< 0.001	0.12	< 0.001	0.12	< 0.001
**Dependent var**:	Anger		Anxiety		Collective Angst	
	β	*P*	β	*P*	β	*p*
Perceived control	–0.12	< 0.001	–0.14	< 0.001	–0.07	0.02
χ^2^(df)	27.27(6), *p* < 0.001		59.39(6), *p* < 0.001		34.59 (6), *p* < 0.001	
Comparative fit index	0.97		0.92		0.96	
Standardized root mean square residual	0.03		0.03		0.03	

**TABLE 2:** Indirect associations (standardized parameters)

	Anxiety	Collective angst	Anger
Trust in political institutions, T_1_	–0.04 (*SE* = 0.01)***	–0.02 (*SE* = 0.01)*	–0.03 (*SE* = 0.01)***
Trust in political institutions, T_2_–T_1_	–0.02 (*SE* = 0.01)**	–0.01 (*SE* = 0.01)*	–0.02 (*SE* = 0.01)**
Trust in *super partes* institutions, T_1_	–0.02 (*SE* = 0.01)**	–0.02 (*SE* = 0.01)*	–0.02 (*SE* = 0.01)**
Trust in *super partes* institutions, T_2_–T_1_	–0.02 (*SE* = 0.01)***	–0.01 (*SE* = 0.01), *p* = 0.054	–0.01 (*SE* = 0.01)**
Trust in international institutions, T_1_	–0.03 (*SE* = 0.01)***	–0.01 (*SE* = 0.01)*	–0.02 (*SE* = 0.01)**
Trust in international institutions, T_2_‐T_1_	–0.02 (*SE* = 0.01)**	–0.01 (*SE* = 0.01)*	–0.01 (*SE* = 0.01)**

*** *p* < 0.001;

** *p* < 0.01;

* *p* < 0.05.

## DISCUSSION

Thus far, several studies have documented that in many countries, the COVID‐19 pandemic triggered an increase in the public's institutional trust (e.g., Bol et al., 2020). This evidence can be traced to the framework of the rally effect phenomenon, supporting that rally effects are limited neither to endogenous threats (war and terrorist attacks) nor to a specific socio‐political context (the United States), or critical decision‐making institution (the government). We anticipated that, beyond the collective function of restoring collective well‐being and promoting compliance with the unpopular measures taken to prevent the spread of the virus (Lazarus et al., 2020), increased institutional trust may fulfil the individual functions of compensating for weakened perceived control over one's life and limiting negative emotions. Our predictions were borne out: Increased trust in political and *super partes* institutions served the psychological function of successfully coping with a dramatic event, promoting participants’ perceived control over their lives.

The perception of control over one's life and surrounding social environment is essential to ensure the meaning and worth of individual acts (Janoff‐Bulman, 1992). For this reason, we observed that perceived control was associated with less negative emotions experienced by participants 6 months later. These mediated effects were found on measures of three emotions (anxiety, anger, and collective angst) that are typical consequences of exogenous shocks, extending the generalizability of the present findings. Although our study is limited in defining the causal mechanism only in terms of temporal sequence, our confidence in these results is enhanced by the longitudinal design of our study, which allowed us to test a sequential model in which independent, mediating, and dependent variables were measured at different points in time. Therefore, our analyses expand previous findings of rally effects, showing that increased trust in response to an exogenous crisis (the COVID‐19 pandemic) also fulfilled an individual function of promoting individual well‐being. This new evidence broadens Parker's (1995) classic idea that rally effects “invoke feelings of allegiance toward *national political institutions and policies*” (p. 526, emphasis added), not promoting mere patriotism or presidential popularity but also support for the entire institutional system at large.

Our results with regard to trust in international institutions require specific reasoning. While trust in international institutions did not increase between the pre‐pandemic and first pandemic waves across our whole sample, it of course rose for some participants, who benefited from this increase. On the one hand, the rally effects elicited by the COVID‐19 pandemic did not transcend the boundaries of Italy, leading instead to a decrease of trust in international institutions. This is consistent with the idea that in the struggle against COVID‐19, the perception of increasing difficulty in policy coordination across international institutions has favored the spread of a common feeling of “every country for itself” in response to a risk that is common and global (Maffettone and Oldani, 2020). However, this decrease was not generalized. Even the individual increase of trust in international institutions was found to be functional in terms of compensatory control, reflecting higher individual well‐being via the mediation of perceived control over one's life. Future research could establish the generality of these effects across a range of different institutions by conducting cross‐national survey research.

As suggested by Lambert et al. (2010), studies like this one highlight the power of the situation. Indeed, rally effects represent very powerful situation‐specific shifts in public opinion, and our findings confirm that situational threats like the COVID‐19 pandemic can trigger rapid changes in political attitudes. Previous studies have shown that, over time, rally effects weaken because political entrepreneurs come to the foreground and pre‐existing political identifications are reactivated. For example, the rally effects observed in response to the Gulf War lasted for about 10 months (Parker, 1995). In light of this, an interesting challenge for future research would be to investigate whether and for how long these positive effects can last–in other words, the degree to which the individual coping function of institutional trust that we detected is enduring. After years of constant increase, votes for Italian populist parties decreased in the months following the onset of the COVID‐19 pandemic (Russo et al., 2021). This is consistent with the rally effect we have documented because an anti‐institution attitude is at the core of populist orientations (e.g., Kriesi and Pappas, 2015). An integration of Russo et al.'s study (2021) and this research could be interesting in order to analyze longitudinally whether this anti‐populist shift has the function of fostering people's individual well‐being. Even before performing these extensions, however, we believe that we have significantly contributed to the literature on rally effects, showing some previously unknown nuances and functions.

## ACKNOWLEDGEMENTS

Open Access Funding provided by Universita degli Studi di Bologna within the CRUI‐CARE Agreement.

## Supporting information

Table 1. Descriptive statistics and bivariate correlations between study variables
