# Supplementary material for: Coping with the COVID‐19 pandemic through institutional trust: Rally effects, compensatory control, and emotions
Source: Soc Sci Q. 2021 Aug 19;102(5):2360–7. doi: 10.1111/ssqu.13002 (PMC8447168; doi:10.1111/ssqu.13002)
Supplement: Supplementary file 1 — Table 1. Descriptive statistics and bivariate correlations between study variables [file SSQU-102-2360-s001.docx]

**SUPPLEMENTARY APPENDIX**

Table 1. Descriptive statistics and bivariate correlations between study variables

|  | Mean(*SD*) | 2. | 3. | 4. | 5. | 6. | 7. | 8. | 9. | 10. |
| --- | --- | --- | --- | --- | --- | --- | --- | --- | --- | --- |
| 1. Trust in political institutions, T_1_ | .38(.20) | .65*** | .63*** | -.38*** | -.22*** | -.12*** | .21*** | -.02 | -.11*** | -.03 |
| 2. Trust in *super partes* institutions, T_1_ | .57(.21) | - | .67*** | -.18*** | -.35*** | -.18*** | .13*** | .01 | -.14*** | .03 |
| 3. Trust in international institutions, T_1_ | .54(.23) |  | - | -.11*** | -.16*** | -.37*** | .14*** | .07* | -.09** | .01 |
| 4. ΔTrust in political institutions | .03(.18) |  |  | - | .62*** | .50*** | .04 | .07* | .03 | .05 |
| 5. ΔTrust in *super partes* institutions | .01(.17) |  |  |  | - | .53*** | .06 | .08* | .01 | .05 |
| 6. ΔTrust in international institutions | -.11(.20) |  |  |  |  | - | .05 | -.03 | .01 | -.06 |
| 7. Perceived control | .74(1.81) |  |  |  |  |  | - | -.14*** | -.12*** | -.07* |
| 8. Anxiety | 2.88(.79) |  |  |  |  |  |  | - | .53*** | .43*** |
| 9. Anger | 2.55(.82) |  |  |  |  |  |  |  | - | .28*** |
| 10. Collective angst | 3.14(.71) |  |  |  |  |  |  |  |  | - |

*Note*. ****p* < .001. ***p* < .01. **p* < .05.
